# Mortality of Philadelphia for the Quarter Ending January 1, 1857, and a General Summary for the Year 1856

**Published:** 1857-03

**Authors:** Wilson Jewell


ART. II.—*Mortality of Philadelphia, for October, November, and December,* 1856; *collated from the Health Office record.* By WILSON JEWELL, M.D.

THE mortality on record for the last quarter of the year 1856, amounts to 2982. This number of deaths includes a period of ninety-eight days, from September 27th, to January 3d, 1857, and averages thirty and a half deaths per day. The mortality for this quarter exceeds that of the same quarter of 1855 by 811, or more than 15 per cent.

The still born for the quarter amounted to 145; while the deaths from external and other adventitious causes are set down at 331; in all 476: thus reducing the deaths from recorded diseases to 2506.

The deaths among males were 1489; while those among females numbered 1493. In this instance the excess is with the female sex.

The deaths among children under ten years of age amounted to 1601 (exclusive of still born), equivalent to 56·43 per cent.

The mortality from "endemics" amounted to 861; more than 23 per cent. over those for the fourth quarter of 1855.

Of the diseases under this head, scarlet fever furnishes 438 deaths: more than one-half the whole number. This disease has been extensively prevalent during the entire quarter; and from the number of deaths, appears to have been unusually fatal. Almost every ward throughout the entire city has suffered more or less from its ravages; although the western and northern sections have been more severely visited. The period of infancy which appears to have suffered more than any other, was that between two and five years. Four hundred and eighteen of the deaths were in children under ten years of age. Measles furnished 55 of the deaths under this head. This exanthem is on the increase.

The "nervous" system contributed 429 of the deaths for the quarter; being rather more than one-seventh of the whole mortality. Convulsions in children furnished 142, of which number 80 occurred in children under one year old.

The "organs of respiration" are charged with 661 of the deaths. Of these, consumption of the lungs makes up 357, more than one-half, and inflammation of the lungs, 118.

By a comparison of the deaths of this quarter with those under the several heads for the fourth quarter of 1855, it will be found that in every instance, there has been an increase, with the exception of still born, in which there will be found a decrease.

The fourth or last table gives the deaths for each week, and shows that the highest number of deaths in any one week was 269, between the 20th and 27th of December. The lowest in any one week was 166, between the 11th and 18th of October.

The deaths among children, or those under twenty years of age, were 1864, or 25 per cent. more than those over twenty, which amounted to 1118.

### TABLE No. 1.

*Deaths for the Fourth Quarter of the Year* 1856, *classified.*

| | Oct. | Nov | Dec. | Male. O. | Male. N. | Male. D. | Female. O. | Female. N. | Female. D. | Total. |
|---|---|---|---|---|---|---|---|---|---|---|
| 1. *Endemic and Contagious Diseases,* Zymotic or Epidemic, . . . . . | 222 | 253 | 386 | 102 | 103 | 191 | 120 | 150 | 195 | 861 |
| 2. *Uncertain or General Seat,* Sporadic Diseases, . . . . . . | 161 | 133 | 121 | 83 | 65 | 66 | 78 | 68 | 55 | 415 |
| 3. Nervous System, . . . . . . . | 141 | 117 | 171 | 82 | 60 | 86 | 59 | 57 | 85 | 429 |
| 4. Organs of Respiration, . . . . | 218 | 176 | 267 | 105 | 88 | 137 | 113 | 88 | 130 | 661 |
| 5. " Circulation, . . . . | 14 | 22 | 28 | 8 | 10 | 17 | 6 | 12 | 11 | 64 |
| 6. Digestive Organs, . . . . . . . | 36 | 36 | 63 | 14 | 21 | 28 | 22 | 15 | 35 | 135 |
| 7. Urinary " . . . . . . . | 3 | 2 | 7 | 2 | 1 | 7 | 1 | 1 | | 12 |
| 8. Organs of Generation, . . . . | 4 | 6 | 8 | | | | 4 | 6 | 8 | 18 |
| 9. " Locomotion, . . . . | 3 | 2 | 6 | 2 | 1 | 3 | 1 | 1 | 3 | 11 |
| 10. Integumentary System, . . . . | 1 | 2 | 2 | 1 | 1 | 1 | | 1 | 1 | 5 |
| 11. Old Age, . . . . . . . . . . | 16 | 10 | 14 | 7 | 2 | 4 | 9 | 8 | 10 | 40 |
| 12. External Causes, . . . . . . . | 41 | 41 | 25 | 28 | 22 | 15 | 13 | 19 | 10 | 107 |
| Still Born, . . . . . . . . . | 37 | 38 | 70 | 18 | 23 | 37 | 19 | 15 | 33 | 145 |
| Unknown, . . . . . . . . . . | 23 | 20 | 36 | 16 | 12 | 20 | 7 | 8 | 16 | 79 |
| | 920 | 858 | 1204 | 468 | 409 | 612 | 452 | 449 | 592 | 2982 |

### TABLE No. 2.

1. *Endemic and Contagious diseases—Zymotic or Epidemic.*

| | Male. | Female. | Under 1 yr. | 1 to 2. | 2 to 5. | 5 to 10. | 10 to 15. | 15 to 20. | 20 to 30. | 30 to 40. | 40 to 50. | 50 to 60. | 60 to 70. | 70 to 80. | 80 to 90. | 90 to 100. | 100 to 110. | Total. |
|---|---|---|---|---|---|---|---|---|---|---|---|---|---|---|---|---|---|---|
| Cholera, . . . . . | | 1 | | | | | | | 1 | | | | | | | | | 1 |
| " Infantum, . | 9 | 3 | 7 | 5 | | | | | | | | | | | | | | 12 |
| " Morbus, . . | 2 | 1 | | | | 1 | | | | | | 2 | | | | | | 3 |
| Croup, . . . . . | 50 | 48 | 18 | 21 | 45 | 13 | | | | | | 1 | | | | | | 98 |
| Diarrhœa, . . . . | 6 | 14 | 6 | 1 | 1 | | | | 2 | | 4 | 1 | 1 | 3 | 1 | | | 20 |
| Dysentery, . . . . | 20 | 19 | 5 | 6 | 2 | 1 | | | 3 | 4 | 5 | 6 | 2 | 4 | 1 | | | 39 |
| Erysipelas, . . . . | 15 | 12 | 9 | 1 | 2 | | | | 1 | 2 | 4 | 3 | 1 | 3 | 1 | | | 27 |
| Fever, . . . . . | 1 | 3 | | | | | 1 | | 2 | | | | | | | 1 | | 4 |
| " Bilious, . . . | 4 | 3 | | | | | 1 | | 2 | 2 | 1 | | | 1 | | | | 7 |
| " Congestive, . . | | 2 | | 1 | | 1 | | | | | | | | | | | | 2 |
| " Intermittent, . | 1 | 7 | 1 | 1 | 2 | | | | 1 | | | | | 2 | 1 | | | 8 |
| " Nervous, . . | 1 | 2 | | | | 2 | | | 1 | | | | | | | | | 3 |
| " Remittent, . . | 2 | 4 | 1 | | 1 | 1 | | | | | 1 | | 1 | 1 | | | | 6 |
| " Scarlet, . . . | 200 | 238 | 52 | 68 | 199 | 99 | 12 | 3 | 3 | 2 | | | | | | | | 438 |
| " Typhoid, . . | 37 | 43 | 1 | 3 | 5 | 6 | 3 | 7 | 18 | 17 | 7 | 6 | 4 | 3 | | | | 80 |
| " Typhus, . . . | 8 | 5 | | | | 1 | | 1 | 9 | 1 | 1 | | | | | | | 13 |
| " " Icterodes, | 1 | | | | | | | | 1 | | | | | | | | | 1 |
| Hooping-Cough, . . | 3 | 11 | 7 | 3 | 3 | 1 | | | | | | | | | | | | 14 |
| Measles, . . . . . | 22 | 33 | 13 | 14 | 19 | 7 | | 1 | 1 | | | | | | | | | 55 |
| Small-Pox, . . . . | 11 | 14 | 6 | 3 | 3 | 5 | 2 | 1 | 2 | 2 | | | 1 | | | | | 25 |
| Syphilis, . . . . . | 1 | | | 1 | | | | | | | | | | | | | | 1 |
| Varioloid, . . . . | 2 | 2 | | 2 | | 1 | 1 | | | | | | | | | | | 4 |
| | 396 | 465 | 126 | 130 | 282 | 139 | 20 | 13 | 47 | 30 | 23 | 19 | 10 | 17 | 4 | 1 | | 861 |

2. *Uncertain or General Seat—Sporadic Diseases.*

| | Male. | Female. | Under 1 yr. | 1 to 2. | 2 to 5. | 5 to 10. | 10 to 15. | 15 to 20. | 20 to 30. | 30 to 40. | 40 to 50. | 50 to 60. | 60 to 70. | 70 to 80. | 80 to 90. | 90 to 100. | 100 to 110. | Total. |
|---|---|---|---|---|---|---|---|---|---|---|---|---|---|---|---|---|---|---|
| Abscess, . . . . . . . | 5 | 6 | | 2 | 1 | | | | 2 | | 3 | 2 | 1 | | | | | 11 |
| " Psoas, . . . . . | 1 | | | | | | | | 1 | | | | | | | | | 1 |
| Anasarca, . . . . . . | 2 | | | | 2 | | | | | | | | | | | | | 2 |
| Cancer, . . . . . . . | 2 | 19 | 1 | 1 | | | | | 2 | 2 | 3 | 4 | 7 | 1 | | | | 21 |
| " Breast, . . . . . | | 1 | | | | | | | | | | 1 | | | | | | 1 |
| Congestion, . . . . . . | 1 | 1 | 1 | | | | | | | | | 1 | | | | | | 2 |
| Cyanosis, . . . . . . . | 10 | 4 | 12 | | | 2 | | | | | | | | | | | | 14 |
| Debility, . . . . . . . | 54 | 45 | 47 | 3 | 3 | 2 | | | | 1 | 2 | 4 | 8 | 14 | 12 | 3 | | 99 |
| Dropsy, . . . . . . . | 43 | 32 | 2 | 3 | 16 | 12 | 3 | 1 | 5 | 7 | 4 | 7 | 8 | 6 | 1 | | | 75 |
| Fungus Hæmatodes, . . | | 1 | | | | | | | | | 1 | | | | | | | 1 |
| Gangrene, . . . . . . | 3 | 1 | | | | | | 1 | | | 1 | 1 | 1 | | | | | 4 |
| Gout, . . . . . . . . | 2 | | | | | | | | | | 1 | 1 | | | | | | 2 |
| Hemorrhage, . . . . . | 3 | 2 | 1 | | | 1 | | | | 2 | 1 | | | | | | | 5 |
| Hydrophobia, . . . . . | 1 | | | | | 1 | | | | | | | | | | | | 1 |
| Inanition, . . . . . . | 11 | 9 | 14 | 1 | 1 | | | | | | | 1 | | 2 | 1 | | | 20 |
| Inflammation, . . . . . | 1 | 3 | 3 | | | 1 | | | | | | | | | | | | 4 |
| Malformation, . . . . . | 2 | 4 | 6 | | | | | | | | | | | | | | | 6 |
| Marasmus, . . . . . . | 51 | 54 | 50 | 31 | 10 | 4 | | | 2 | 2 | 1 | 1 | 2 | 2 | | | | 105 |
| Mortification, . . . . . | | 1 | | | | | | | | 1 | | | | | | | | 1 |
| Rachitis, . . . . . . . | 3 | | | 1 | 1 | 1 | | | | | | | | | | | | 3 |
| Scirrhus, . . . . . . . | | 2 | | | | | | | | | 2 | | | | | | | 2 |
| Scrofula, . . . . . . . | 7 | 6 | 3 | 3 | 2 | 1 | | | 1 | 1 | | 1 | | 1 | | | | 13 |
| Sore Throat, . . . . . | 2 | 1 | 1 | | 2 | | | | | | | | | | | | | 3 |
| Tabes Mesenterica, . . | 7 | 3 | 3 | 4 | 1 | | | | 1 | | 1 | | | | | | | 10 |
| Tumor, . . . . . . . | | 4 | | | | | | | | | 2 | | 2 | | | | | 4 |
| Ulceration of Throat, . . | 3 | 2 | 1 | | | 4 | | | | | | | | | | | | 5 |
| | 214 | 201 | 145 | 49 | 39 | 29 | 3 | 2 | 14 | 16 | 22 | 24 | 29 | 26 | 14 | 3 | | 415 |

3. *Nervous Diseases.*

| | Male. | Female. | Under 1 yr. | 1 to 2. | 2 to 5. | 5 to 10. | 10 to 15. | 15 to 20. | 20 to 30. | 30 to 40. | 40 to 50. | 50 to 60. | 60 to 70. | 70 to 80. | 80 to 90. | 90 to 100. | 100 to 110. | Total. |
|---|---|---|---|---|---|---|---|---|---|---|---|---|---|---|---|---|---|---|
| Apoplexy, . . . . . . | 19 | 14 | | | | | | | 2 | 7 | 6 | 7 | 8 | 2 | 1 | | | 33 |
| Coma, . . . . . . . . | | 1 | | | | | | | | 1 | | | | | | | | 1 |
| Concussion of Brain, . . | 2 | | | | | | | | 1 | | | | | 1 | | | | 2 |
| Congestion " . . | 21 | 16 | 12 | 3 | 13 | | | 1 | 2 | 1 | 3 | 2 | | | | | | 37 |
| Compression " . . | 1 | 1 | | | | | | | | | 1 | 1 | | | | | | 2 |
| Convulsions, . . . . . . | 70 | 72 | 80 | 27 | 18 | 4 | 1 | 2 | 2 | 4 | 3 | | | 1 | | | | 142 |
| Cramp, . . . . . . . | 3 | | | 1 | 1 | | | 1 | | | | | | | | | | 3 |
| Disease of Brain, . . . | 14 | 9 | 6 | 3 | 4 | | 2 | 1 | 3 | 1 | 2 | | 1 | | | | | 23 |
| Dropsy " . . . . | 21 | 19 | 14 | 10 | 10 | 5 | | | | | 1 | | | | | | | 40 |
| Effusion " . . . . | 12 | 7 | 5 | 5 | 3 | 1 | | 1 | | 2 | | 1 | 1 | | | | | 19 |
| Epilepsy, . . . . . . . | 2 | 1 | | | | | 1 | | 1 | | | 1 | | | | | | 3 |
| Inflammation of Brain, . | 34 | 35 | 26 | 9 | 17 | 10 | 1 | | 3 | 1 | | 1 | | 1 | | | | 69 |
| Mania, . . . . . . . | 1 | 3 | | | | | | | | 1 | 2 | 1 | | | | | | 4 |
| Mania-a-Potu, . . . . | 12 | 5 | | | | | | | 2 | 8 | 2 | 4 | | 1 | | | | 17 |
| Palsy, . . . . . . . . | 7 | 11 | | 1 | | | | 1 | | 3 | 4 | 1 | 2 | 4 | 2 | | | 18 |
| Softening of Brain, . . | 6 | 6 | 2 | | 2 | | | 1 | | 2 | 2 | 1 | 1 | 1 | | | | 12 |
| Tetanus, . . . . . . . | 3 | 1 | 1 | | | | | 1 | 1 | | 1 | | | | | | | 4 |
| | 228 | 201 | 146 | 59 | 68 | 20 | 5 | 9 | 17 | 31 | 27 | 20 | 13 | 11 | 3 | | | 429 |

### 4. *Organs of Respiration.*

| | Male. | Female. | Under 1 yr. | 1 to 2. | 2 to 5. | 5 to 10. | 10 to 15. | 15 to 20. | 20 to 30. | 30 to 40. | 40 to 50. | 50 to 60. | 60 to 70. | 70 to 80. | 80 to 90. | 90 to 100. | 100 to 110. | Total. |
|---|---|---|---|---|---|---|---|---|---|---|---|---|---|---|---|---|---|---|
| Asthma, . . . . . . | 2 | 1 | | | | | | | 1 | | 1 | | | 1 | | | | 3 |
| Congestion of Lungs, . | 12 | 16 | 7 | 3 | 6 | 2 | | | 2 | 1 | 1 | 2 | 2 | 1 | 1 | | | 28 |
| Consumption of Lungs, . | 174 | 183 | 6 | 10 | 11 | 7 | 4 | 31 | 104 | 85 | 44 | 28 | 21 | 5 | 1 | | | 357 |
| Disease of Chest, . . . | 1 | | | | 1 | | | | | | | | | | | | | 1 |
| Disease of Lungs, . . | 7 | 2 | 3 | | | | | | | | 2 | 1 | | 2 | | 1 | | 9 |
| Dropsy of Chest, . . . | 12 | 8 | | 1 | 9 | 4 | | | 2 | | | 2 | 1 | 1 | | | | 20 |
| Effusion on Chest, . . | 1 | | | | 1 | | | | | | | | | | | | | 1 |
| " Lungs, . . | 1 | 1 | | | | | | | | | | | 1 | | 1 | | | 2 |
| Hemorrhage from Lungs, | 6 | 7 | 1 | | | | | 1 | 3 | 5 | | 1 | 2 | | | | | 13 |
| Inflammation of Bronchi, | 40 | 39 | 33 | 18 | 15 | | | | 2 | 2 | | 1 | 2 | 4 | 2 | | | 79 |
| " Chest, . | 1 | 1 | | | 1 | | | | | | | | 1 | | | | | 2 |
| " Larynx, | 5 | 7 | 1 | 1 | 4 | 2 | | | 2 | 1 | | 1 | | | | | | 12 |
| " Lungs, . | 58 | 60 | 29 | 17 | 35 | 5 | 3 | 3 | 6 | 5 | 5 | 5 | 1 | 2 | 2 | | | 118 |
| " Pleura, | 5 | 2 | | | | | | | 2 | 1 | 2 | | 2 | | | | | 7 |
| " Trachea, | 3 | 2 | | 1 | 3 | | | | 1 | | | | | | | | | 5 |
| " Tonsils, | 1 | 2 | 2 | | | | | | | 1 | | | | | | | | 3 |
| Palsy of Lungs, . . . | 1 | | | | 1 | | | | | | | | | | | | | 1 |
| | 330 | 331 | 82 | 51 | 87 | 20 | 7 | 35 | 125 | 101 | 55 | 41 | 33 | 16 | 7 | 1 | | 661 |

### 5. *Organs of Circulation.*

| | Male. | Female. | Under 1 yr. | 1 to 2. | 2 to 5. | 5 to 10. | 10 to 15. | 15 to 20. | 20 to 30. | 30 to 40. | 40 to 50. | 50 to 60. | 60 to 70. | 70 to 80. | 80 to 90. | 90 to 100. | 100 to 110. | Total. |
|---|---|---|---|---|---|---|---|---|---|---|---|---|---|---|---|---|---|---|
| Anæmia, . . . . . . . . | 1 | 1 | | 1 | | | | | | | 1 | | | | | | | 2 |
| Aneurism, . . . . . . . . | 3 | | | | | | | | 1 | 1 | | 1 | | | | | | 3 |
| Angina Pectoris, . . . . . | 1 | | | | | | | | | | 1 | | | | | | | 1 |
| Disease of Heart, . . . . | 20 | 20 | 4 | 2 | 1 | 2 | | 2 | 5 | 4 | 2 | 5 | 7 | 4 | 2 | | | 40 |
| Dropsy of Heart, . . . . . | 2 | 3 | | | 1 | 1 | | | | 1 | | | 2 | | | | | 5 |
| Enlargement of Heart, . . . | 5 | 2 | | | | 1 | 1 | | | 1 | 1 | | 2 | 1 | | | | 7 |
| Inflammation of Heart, . . | 2 | 1 | | | | 1 | | | | 2 | | | | | | | | 3 |
| Ossification of Heart, . . . | | 1 | | | | | | | | 1 | | | | | | | | 1 |
| " Pulmonary Arteries, | | 1 | | | | | | | | | | | | 1 | | | | 1 |
| Rupture of Heart, . . . . | 1 | | | | | | | | 1 | | | | | | | | | 1 |
| | 35 | 29 | 4 | 3 | 2 | 5 | 1 | 2 | 7 | 10 | 5 | 6 | 11 | 6 | 2 | | | 64 |

6. *Digestive Organs.*

| | Male. | Female. | Under 1 yr. | 1 to 2. | 2 to 5. | 5 to 10. | 10 to 15. | 15 to 20. | 20 to 30. | 30 to 40. | 40 to 50. | 50 to 60. | 60 to 70. | 70 to 80. | 80 to 90. | 90 to 100. | 100 to 110. | Total. |
|---|---|---|---|---|---|---|---|---|---|---|---|---|---|---|---|---|---|---|
| Abdominal Dropsy, . . . | | 1 | | | 1 | | | | | | | | | | | | | 1 |
| Abscess of Liver, . . . . | 1 | | | | | | | | | | 1 | | | | | | | 1 |
| Cancer of Rectum, . . . . | | 1 | | | | | | | | | 1 | | | | | | | 1 |
| " Stomach, . . . | 2 | | | | | | | | | | 1 | 1 | | | | | | 2 |
| Cancrum Oris, . . . . . | | 1 | | | 1 | | | | | | | | | | | | | 1 |
| Cirrhosis of Liver, . . . . | 1 | | | | | | | | | | | 1 | | | | | | 1 |
| Colic, . . . . . . . . . | 3 | 3 | 1 | | | | | 1 | | 1 | | 2 | 1 | | | | | 6 |
| Congestion of Liver, . . . | 1 | | | | | | | | | | 1 | | | | | | | 1 |
| Constipation, . . . . . . | 1 | | | | | | | | | | 1 | | | | | | | 1 |
| Cramp of Stomach, . . . | 1 | | | | | | | | | | 1 | | | | | | | 1 |
| Disease of Liver, . . . . | | 4 | 1 | | | | | | | | 1 | 1 | | 1 | | | | 4 |
| " Stomach & Bowels, | 4 | 5 | 5 | | | 1 | | | | | | | 1 | 2 | | | | 9 |
| Enlargement of Liver, . . | | 1 | | | 1 | | | | | | | | | | | | | 1 |
| Hem'age from Stom. & Bow., | 3 | 3 | | | | | | | 3 | | 1 | 1 | | 1 | | | | 6 |
| " Umbilicus, . | | 1 | | | | | | 1 | | | | | | | | | | 1 |
| Hernia, . . . . . . . . | 1 | | 1 | | | | | | | | | | | | | | | 1 |
| " Strangulated, . . | 1 | 3 | | | | | | | | 1 | | 1 | 2 | | | | | 4 |
| Ileus, . . . . . . . . . | 1 | | | | | | | | | 1 | | | | | | | | 1 |
| Inflamm'n of Liver, . . . | 4 | 3 | | 1 | | | 2 | | 1 | | | 2 | | 1 | | | | 7 |
| " Peritoneum, . | 9 | 12 | 1 | 1 | 2 | | 3 | 1 | 7 | 4 | 1 | 1 | | | | | | 21 |
| " Stom. and Bow., | 22 | 26 | 7 | 4 | 5 | 3 | 2 | 2 | 5 | 6 | 6 | 3 | 4 | 1 | | | | 48 |
| Intussusception, . . . . | 1 | | | | | | | | | | | 1 | | | | | | 1 |
| Jaundice, . . . . . . . . | 1 | 2 | 1 | | | | | | | | | 1 | | | 1 | | | 3 |
| Perforation of Intestines, . | | 1 | | | | 1 | | | | | | | | | | | | 1 |
| Stricture of Bowels, . . . | 1 | | 1 | | | | | | | | | | | | | | | 1 |
| Teething, . . . . . . . . | 2 | 2 | 3 | 1 | | | | | | | | | | | | | | 4 |
| Ulceration of Stom. and Bow., | 2 | 3 | | | | | | | | | 3 | | 2 | | | | | 5 |
| Worms, . . . . . . . . | 1 | | 1 | | | | | | | | | | | | | | | 1 |
| | 63 | 72 | 22 | 7 | 10 | 5 | 7 | 5 | 16 | 13 | 18 | 15 | 10 | 6 | 1 | | | 135 |

7. *Urinary Organs.*

| | Male. | Female. | Under 1 yr. | 1 to 2. | 2 to 5. | 5 to 10. | 10 to 15. | 15 to 20. | 20 to 30. | 30 to 40. | 40 to 50. | 50 to 60. | 60 to 70. | 70 to 80. | 80 to 90. | 90 to 100. | 100 to 110. | Total. |
|---|---|---|---|---|---|---|---|---|---|---|---|---|---|---|---|---|---|---|
| Albuminuria, . . . . . . . | 3 | | | | | | | | 1 | | 2 | | | | | | | 3 |
| Anuria, . . . . . . . . . | | 1 | 1 | | | | | | | | | | | | | | | 1 |
| Disease of Bladder, . . . . | | 1 | | | 1 | | | | | | | | | | | | | 1 |
| " Kidneys, . . . . | 5 | | | | | | | | 1 | | 1 | 2 | 1 | | | | | 5 |
| " Urinary Organs, . | 1 | | | | | | | | | | | | 1 | | | | | 1 |
| Inflammation of Bladder, . . | 1 | | | | | | | | | | | | 1 | | | | | 1 |
| | 10 | 2 | 1 | | 1 | | | | 2 | | 3 | 2 | 3 | | | | | 12 |

8. *Organs of Generation.*

| | Male. | Female. | Under 1 yr. | 1 to 2. | 2 to 5. | 5 to 10. | 10 to 15. | 15 to 20. | 20 to 30. | 30 to 40. | 40 to 50. | 50 to 60. | 60 to 70. | 70 to 80. | 80 to 90. | 90 to 100. | 100 to 110. | Total. |
|---|---|---|---|---|---|---|---|---|---|---|---|---|---|---|---|---|---|---|
| Cancer of Uterus, . . . . | | 2 | | | | | | | | | | 2 | | | | | | 2 |
| Convulsions, Puerperal, . . | | 1 | | | | | | | | 1 | | | | | | | | 1 |
| Disease of Ovaries, . . . . | | 1 | | | | | | | | 1 | | | | | | | | 1 |
| Hemorrhage from Uterus, . . | | 1 | | | | | | | | 1 | | | | | | | | 1 |
| Inflammation of Uterus, . . | | 4 | | | | | | | 2 | 2 | | | | | | | | 4 |
| Puerperal Fever, . . . . | | 8 | | | | | | | 2 | 6 | | | | | | | | 8 |
| Rupture of Uterus, . . . . | | 1 | | | | | | | | 1 | | | | | | | | 1 |
| | | 18 | | | | | | | 4 | 12 | | 2 | | | | | | 18 |

9. *Organs of Locomotion.*

| | Male. | Female. | Under 1 yr. | 1 to 2. | 2 to 5. | 5 to 10. | 10 to 15. | 15 to 20. | 20 to 30. | 30 to 40. | 40 to 50. | 50 to 60. | 60 to 70. | 70 to 80. | 80 to 90. | 90 to 100. | 100 to 110. | Total. |
|---|---|---|---|---|---|---|---|---|---|---|---|---|---|---|---|---|---|---|
| Caries, . . . . . . . . | 1 | 1 | | | | | | | | 2 | | | | | | | | 2 |
| Disease of Spine, . . . . . | 2 | | | | | 1 | | | | | | 1 | | | | | | 2 |
| Rheumatism, . . . . . . | 2 | 4 | | | | 1 | | | | | 1 | 2 | 2 | | | | | 6 |
| Spina Bifida, . . . . . . | 1 | | 1 | | | | | | | | | | | | | | | 1 |
| | 6 | 5 | 1 | | | 2 | | | | 2 | 1 | 3 | 2 | | | | | 11 |

10. *Integumentary System.*

| | Male. | Female. | Under 1 yr. | 1 to 2. | 2 to 5. | 5 to 10. | 10 to 15. | 15 to 20. | 20 to 30. | 30 to 40. | 40 to 50. | 50 to 60. | 60 to 70. | 70 to 80. | 80 to 90. | 90 to 100. | 100 to 110. | Total. |
|---|---|---|---|---|---|---|---|---|---|---|---|---|---|---|---|---|---|---|
| Carbuncle, . . . . . . . | | 1 | | | | | | | 1 | | | | | | | | | 1 |
| Lupus Exedens, . . . . . | 1 | | | | | | | | | | | 1 | | | | | | 1 |
| Purpura Hemorrhagica, . . | 2 | 1 | 1 | 1 | 1 | | | | | | | | | | | | | 3 |
| | 3 | 2 | 1 | 1 | 1 | | | | 1 | | | 1 | | | | | | 5 |

11. *Old Age.*

| | | | | | | | | | | | | | | | | | | |
|---|---|---|---|---|---|---|---|---|---|---|---|---|---|---|---|---|---|---|
| Old Age, . . . . . . . . | 13 | 27 | | | | | | | | | | | 2 | 8 | 22 | 7 | 1 | 40 |

12. *External Causes.*

| | Male. | Female. | Under 1 yr. | 1 to 2. | 2 to 5. | 5 to 10. | 10 to 15. | 15 to 20. | 20 to 30. | 30 to 40. | 40 to 50. | 50 to 60. | 60 to 70. | 70 to 80. | 80 to 90. | 90 to 100. | 100 to 110. | Total. |
|---|---|---|---|---|---|---|---|---|---|---|---|---|---|---|---|---|---|---|
| Asphyxia, . . . . . . . | 3 | 8 | 9 | | | | | | | 2 | | | | | | | | 11 |
| Burns, . . . . . . . . . | 5 | 16 | 2 | | 6 | 6 | | | 3 | 1 | 2 | 1 | | | | | | 21 |
| Casualties, . . . . . . . | 24 | 7 | 2 | 1 | | 1 | 3 | 1 | 5 | 10 | 5 | 2 | 1 | | | | | 31 |
| Drowned, . . . . . . . . | 10 | 2 | | | | 1 | | 3 | 4 | 2 | 1 | 1 | | | | | | 12 |
| Exhaustion, . . . . . . . | 1 | 1 | 1 | | | | | | | | | | 1 | | | | | 2 |
| Fracture of Skull, . . . . | 1 | | | | | | | | 1 | | | | | | | | | 1 |
| Intemperance, . . . . . | 1 | 4 | | | | | | | 3 | 2 | | | | | | | | 5 |
| Poisoning, . . . . . . . | 2 | | | | | | | | | | 1 | 1 | | | | | | 2 |
| Suffocation, . . . . . . . | 3 | 2 | | | 2 | 1 | | | | 1 | | 1 | | | | | | 5 |
| Suicide, . . . . . . . . | 11 | 2 | | | | | | 1 | 2 | 3 | 4 | 2 | 1 | | | | | 13 |
| Violence, . . . . . . . . | 4 | | | | | | | 2 | 1 | | 1 | | | | | | | 4 |
| | 65 | 42 | 14 | 1 | 8 | 9 | 3 | 7 | 19 | 21 | 14 | 8 | 3 | | | | | 107 |

| | Male. | Female. | Under 1 yr. | 1 to 2. | 2 to 5. | 5 to 10. | 10 to 15. | 15 to 20. | 20 to 30. | 30 to 40. | 40 to 50. | 50 to 60. | 60 to 70. | 70 to 80. | 80 to 90. | 90 to 100. | 100 to 110. | Total. |
|---|---|---|---|---|---|---|---|---|---|---|---|---|---|---|---|---|---|---|
| Still Born, . . . . . . . | 78 | 67 | 145 | | | | | | | | | | | | | | | 145 |
| Unknown, . . . . . . . | 48 | 31 | 27 | 1 | 3 | | | | 6 | 13 | 9 | 10 | 6 | 3 | | 1 | | 79 |

TABLE No. 3.

*Deaths for the Fourth Quarter of* 1856, *at fifteen distinct periods of life.*

| | |
|---|---|
| Under 1 year, . . . . . . . . . | 569 |
| 1 to 2, . . . . . . . . . | 302 |
| 2 to 5, . . . . . . . . . | 501 |
| 5 to 10, . . . . . . . . | 229 |
| 10 to 15, . . . . . . . . | 46 |
| 15 to 20, . . . . . . . . | 73 |
| 20 to 30, . . . . . . . . | 258 |
| 30 to 40, . . . . . . . . | 249 |
| 40 to 50, . . . . . . . . | 177 |
| 50 to 60, . . . . . . . . | 151 |
| 60 to 70, . . . . . . . . | 122 |
| 70 to 80, . . . . . . . . | 93 |
| 80 to 90, . . . . . . . . | 53 |
| 90 to 100, . . . . . . . . | 13 |
| 100 to 110, . . . . . . . . | 1 |
| | 2837 |
| Still born, . . . . . . . . . | 145 |
| Total, . . . . . . | 2982 |

Of the above deaths there were 122 from the Blockley Almshouse; from the Colored Population, 148; from the Country, 26; from the County Prison, 3; and from the Pennsylvania Hospital, 12, as follows:

| | October. | November. | December. | Total. |
|---|---|---|---|---|
| Almshouse, . . . | 36 | 57 | 29 | 122 |
| Blacks, . . . . | 41 | 65 | 42 | 148 |
| Country, . . . | 10 | 4 | 12 | 26 |
| County Prison, . . | 1 | 1 | 1 | 3 |
| Pennsylvania Hospital, . | 5 | 1 | 6 | 12 |
| | 93 | 128 | 90 | 311 |

TABLE No. 4.

The following table gives the number of deaths for each week during the quarter, together with the sexes, the adults, and children.

| | Male. | Female. | Adults. | Children. | Total. | Monthly Total. |
|---|---|---|---|---|---|---|
| 1856. From Sept. 27th to Oct. 4th, | 104 | 98 | 88 | 114 | 202 | |
| " Oct. 4th to Oct. 11th, | 91 | 109 | 99 | 101 | 200 | |
| " Oct. 11th to Oct. 18th, | 87 | 79 | 70 | 96 | 166 | |
| " Oct. 18th to Oct. 25th, | 88 | 79 | 69 | 98 | 167 | |
| " Oct. 25th to Nov. 1st, | 98 | 87 | 76 | 109 | 185 | 920 |
| " Nov. 1st to Nov. 8th, | 114 | 99 | 82 | 131 | 213 | |
| " Nov. 8th to Nov. 15th, | 109 | 112 | 71 | 150 | 221 | |
| " Nov. 15th to Nov. 22d, | 67 | 125 | 70 | 122 | 192 | |
| " Nov. 22d to Nov. 29th, | 119 | 113 | 81 | 151 | 232 | 858 |
| " Nov. 29th to Dec. 6th, | 132 | 100 | 80 | 152 | 232 | |
| " Dec. 6th to Dec. 13th, | 115 | 118 | 78 | 155 | 233 | |
| " Dec. 13th to Dec. 20th, | 101 | 121 | 75 | 147 | 222 | |
| " Dec. 20th to Dec. 27th, | 148 | 121 | 99 | 170 | 269 | |
| " Dec. 27th to Jan. 3d, 1857, | 116 | 132 | 80 | 168 | 248 | 1204 |
| Totals, | 1489 | 1493 | 1118 | 1864 | 2982 | 2982 |

### ANNUAL MORTALITY OF PHILADELPHIA FOR 1856.

In collecting and arranging the deaths, with their several causes, which have taken place in our city during the past year, as well as in comparing them with the deaths for a series of years, both here and elsewhere, it becomes a cause for regret, that the mortuary record preserved at the Health Office is so barren of interest. An examination of this record will furnish only the name, age, sex, and disease of the deceased, with the name of the physician who gives the certificate of death. The particular residence, the occupation, and the nativity of those who die, are seldom if ever entered upon the face of the certificate, and hence are lost to the statistician, the sanitarian, and the political economist. These items contain facts and information which are indispensable, in order to determine with any degree of accuracy whatever, the sanitary and mortuary condition of all classes of our citizens, or the salubrity or unhealthiness of the several wards or districts of our metropolis.

If, as has been wisely observed, the prosperity of a people is indicated by their degree of public health, and that "public health is to the masses what personal health is to the individual," then it must be obvious, that the importance of having correct statistics of the health of every community is inestimable. But to secure this information, it becomes necessary to know not only the annual death-rate to the population, but to be familiar with those districts which suffer unusual mortality, and all those physical and moral causes which constitute the chief sources for disease and death.

Nothing less than a model system of registration of deaths can possibly answer for the effectual accomplishment of an object so desirable. Until this is secured, our Reports of the Annual Mortality of Philadelphia will be wanting in those essentials which alone can render them truly valuable.

We have faith, however, to believe, that our own State, though at present far behind several others in legislative enactments for the protection of life, will not be willing to remain long in the rear on this question; and that the day is not far distant when we shall witness a marked change—when the authorities, guided by the results of sanitary legislation in other States, will awake to their true condition, and petition for a code of laws to meet those sanitary requirements which otherwise can never be realized: a code of laws, which, when enforced, will secure the blessing of health to thousands, improve the social condition of a large but now too often dependent population, and at the same time lessen sensibly the annual rate of mortality. A well-defined registration law is the first step in this reform.

According to the quarterly tabulated records, which have appeared in the Medical Examiner, the whole mortality in Philadelphia for the year 1856 has been 12,334, as follows:

| | | | |
|---|---|---|---|
| 1st Quarter, . . . | 2810 | 3d Quarter, . . . | 3971 |
| 2d Quarter, . . . | 2571 | 4th Quarter, . . . | 2982 |

This amount shows an increase of 8·23 per cent. over the mortality for 1855.

If the population of our city is 550,000,* the deaths have been one in every 44½, or to each thousand of the population, 22·425.

The average monthly mortality was 1027 $\frac{10}{12}$ths, while the highest number of deaths in any one month, 1796, occurred in July, and the lowest number, 736, in May.

The average deaths per day from Dec. 29th, 1855, to Jan. 3d, 1857, or 370 days, has been 33·33.

The following table designates the deaths during the year, at sixteen different ages:

* By many it is thought to be 600,000.

| | |
|---|---|
| Under 1 year, | 2938 |
| 1 to 2, | 1391 |
| 2 to 5, | 1629 |
| 5 to 10, | 667 |
| 10 to 15, | 228 |
| 15 to 20, | 325 |
| 20 to 30, | 1176 |
| 30 to 40, | 970 |
| 40 to 50, | 686 |
| 50 to 60, | 580 |
| 60 to 70, | 478 |
| 70 to 80, | 390 |
| 80 to 90, | 204 |
| 90 to 100, | 56 |
| 100 to 110, | 3 |
| 110 to 120, | 1 |
| | 11,722 |
| Still born, | 612 |
| Total, | 12,334 |

It will be seen, that this division of deaths, at the several periods of life, as designated, does not include all that have been born, but those only who were born alive. Hence the calculations have been based upon this estimate, and do not embrace the still born, which amounted to 612.

This analytical table exhibits a single fact, that should not go unobserved, viz., the large proportion of deaths during infancy, when compared with the mortality in adult life.

The proportion of deaths in children under one year, is almost double that of any other stage of life, amounting to 2938, or 25 per cent. of the entire mortality.

Those under five years amounted to 5968, nearly equal to 51 per cent. of all the deaths.

Calculating the population of our city under five years of age to be in proximity to 54,000, then the ratio of mortality has been about one in every nine.

The deaths under twenty were 7178, equal to 58·19 per cent. of all the deaths during the year.

From twenty up to one hundred and twenty years, constituting adult life, there were 4544 deaths. Of these, 1176 were between twenty and thirty years. From this period there is a gradual decrease up to extreme old age.

654, or 5·57 per cent. of the annual mortality, had passed the seventieth year; three of whom were centenarians, and one had lived beyond the one hundred and tenth year.

The following tabular statement furnishes a synopsis of those diseases which have been most fatal for the past nine years.

| | 1848 | 1849 | 1850 | 1851 | 1852 | 1853 | 1854 | 1855 | 1856 |
|---|---|---|---|---|---|---|---|---|---|
| Cholera Infantum, . . . | 454 | 582 | 505 | 397 | 329 | 398 | 715 | 566 | 722 |
| Consumption of Lungs, . | 965 | 939 | 907 | 881 | 1204 | 1246 | 1394 | 1327 | 1501 |
| Congestion of Brain, . . | 85 | 91 | 97 | 130 | 120 | 172 | 222 | 175 | 177 |
| Convulsions, . . . . . . | 401 | 415 | 444 | 479 | 499 | 543 | 695 | 626 | 603 |
| Croup, . . . . . . . | 177 | 130 | 143 | 180 | 208 | 303 | 304 | 265 | 268 |
| Diarrhœa, . . . . . | 122 | 225 | 208 | 157 | 156 | 130 | 219 | 177 | 149 |
| Dropsy of Brain, . . . | 220 | 237 | 283 | 245 | 247 | 192 | 299 | 254 | 242 |
| Dysentery, . . . . . | 315 | 578 | 421 | 401 | 558 | 379 | 446 | 266 | 301 |
| Inflammation of Brain, . | 186 | 198 | 218 | 202 | 258 | 157 | 362 | 288 | 329 |
| " " Bronchi, | 172 | 169 | 191 | 175 | 208 | 197 | 243 | 233 | 250 |
| " " Lungs, . | 265 | 273 | 352 | 352 | 444 | 353 | 472 | 442 | 379 |
| Marasmus, . . . . . | 237 | 264 | 217 | 255 | 354 | 376 | 457 | 441 | 484 |
| Scarlet Fever, . . . . | 172 | 242 | 439 | 400 | 433 | 391 | 165 | 163 | 992 |
| Small-Pox, . . . . . | 100 | 152 | 40 | 216 | 426 | 52 | 40 | 281 | 390 |

With but four exceptions, all the diseases enumerated in this table show an increase of deaths over those for the preceding year. Indeed, several of them, as Cholera Infantum, Consumption of the Lungs, Congestion of the Lungs, Marasmus, and Scarlet Fever, present an increase over any year since 1848.

The increase of deaths from Cholera Infantum over those of 1855 is equal to 12·11 per cent.; but compared with the preceding year, 1854, it gives nearly the same aggregate. Consumption of the Lungs furnishes 1501 deaths, or about 12·83 per cent. of the entire mortality, throwing out the still born. To the population, it is as one in every 366½, and to each thousand as 2·72. Throughout the year the mortality from Consumption appears to have had but little variation, every month furnishing its victims to this insatiable disease. The 4th quarter of the year shows the fewest deaths, and the 1st quarter the largest number. During the last nine years, 10,364 deaths have occurred in our city from this disease.

Scarlet Fever contributes 992 of the deaths for the year, rising one-twelfth of the entire mortality, or nearly one-seventh of all the deaths under twenty years of age.

This disease has never prevailed as an epidemic to the same extent in our city, if the mortality is any evidence of its prevalence. In the last three months of the year there were 438 deaths, equal to 4½ deaths per day. It has been with us a subject of inquiry to ascertain what particular sections of our city were more devastated by this terrible epidemic than others; the most reliable information we can secure induces us to believe that Kensington East and West, and those densely inhabited portions of the city between Broad Street and the Schuylkill River, suffered to a very large extent. As usual, the children of the working-classes, and those of the German population, especially in the upper sections of the old Northern Liberty district, suffered severely. At the same time it must be conceded, that the offspring of the better classes, and of our wealthier population, were by no means exempted.

That period of infancy which suffered the most by death was between the ages of two and five years.

For the past eight years, the deaths from Scarlet Fever in this city have numbered 3397.

The deaths from Small-pox have amounted to 390. 76 per cent. of this mortality was in children, or those classed under twenty years of age,—an evidence of the inefficiency of our municipal system for vaccination.

Of the above deaths from Small-pox, 238 took place in the early part of the year, and before the 1st of March, since which date they have been rapidly declining. During the last quarter of the year there were only 25 deaths.

The following tabular statement shows the mortality for the year as classified, together with the rate of percentage to the whole number of deaths, and the number in every thousand of the population, estimating the population as 550,000.

| | Yearly Mortality. | Per cent. of Deaths. | Number per thousand of population. |
|---|---|---|---|
| 1. *Endemic and Contagious Diseases:* | | | |
| Zymotic or Epidemic, . . . . | 3595 | 24·19 | 6·536 |
| 2. *Uncertain or General Seat:* | | | |
| Sporadic Diseases, . . . . . | 1733 | 14·05 | 3·150 |
| 3. Nervous System, . . . . . . . . | 1944 | 15·76 | 3·534 |
| 4. Organs of Respiration, . . . . | 2470 | 20·02 | 4·490 |
| 5. " Circulation, . . . . . . | 267 | 2·16 | 0·480 |
| 6. " Digestion, . . . . . . | 548 | 4·43 | 0·996 |
| 7. Urinary Organs, . . . . . . . . | 51 | 0·41 | 0·092 |
| 8. Organs of Generation, . . . . . | 117 | 0·94 | 0·212 |
| 9. " Locomotion, . . . . | 51 | 0·41 | 0·092 |
| 10. Integumentary System, . . . . | 31 | 0·25 | 0·056 |
| 11. Old Age, . . . . . . . . . . | 172 | 1·39 | 0·313 |
| 12. External Causes, . . . . . . . | 486 | 3·94 | 0·883 |
| Still born, . . . . . . . . . . | 612 | 4·96 | 1·112 |
| Unknown, . . . . . . . . . . | 257 | 2·08 | 0·467 |

From this table it will be seen, that those diseases which properly belong to that class designated "Endemic and Contagious," and by all sanitarians looked upon as preventible or dependent upon causes that are either removable or capable of mitigation by a thorough system of sanitary police, rate higher by nine per cent. than the highest number of deaths in any other classification. The deaths from diseases of the organs of respiration are set down at 2470, equal to 20·02 per cent. of the mortality. Of these deaths, Consumption of the Lungs contributes 1501, or 60 per cent. of the whole, and if deducted therefrom, leaves the mortality less than 1000.

The diseases of the Nervous System give 15·76 per cent., and those of uncertain seat 14·05 per cent.

The Still Born furnish 4·96 per cent. of the mortality, while External Causes contribute 3·94 per cent.

The deaths monthly, and according to the sex, have been as follows:

| Month. | Male. | Female. | Total. |
|---|---|---|---|
| January, | 573 | 533 | 1106 |
| February, | 423 | 438 | 861 |
| March, | 447 | 396 | 843 |
| April, | 548 | 504 | 1052 |
| May, | 357 | 379 | 736 |
| June, | 415 | 368 | 783 |
| July, | 961 | 835 | 1796 |
| August, | 696 | 615 | 1311 |
| September, | 450 | 414 | 864 |
| October, | 468 | 452 | 920 |
| November, | 409 | 449 | 858 |
| December, | 612 | 592 | 1204 |
| | 6359 | 5975 | 12,334 |

The disparity between the deaths in the male and female sexes, as exhibited by this statement, is not as great by one-half as is usually presented. Here, the excess of the male deaths over the females is but 3·11 per cent., while last year, and for several previous years, it ranged over 6 per cent.

During the year, the Almshouse furnished 604 of the deaths, the colored population 605, the Pennsylvania Hospital 46, and the County Prison 14, while 132 are noted, as having been brought into the city for burial, from other places.

The tabulated mortality of our city, which has been compiled from the record, is only an approximation towards the actual number of deaths from disease. Taken in the aggregate, we have credit for a much larger amount than has really existed. Nor is this peculiar to our own city, as we find that in estimating the annual deaths in other large cities, the calculations are generally based on the total of deaths, without particular reference to causes.

A careful analysis of the tables in the present instance, will show that 2167, or 17·56 per cent. of the deaths do not properly belong to the mortality from designated disease, and that, in estimating the comparative health of the city, the deaths from these causes should be deducted. They stand as follows:

| | | | |
|---|---|---|---|
| External Causes, | 486 | Still born, | 612 |
| Old Age, | 172 | Unknown, | 257 |
| From Country, | 132 | Debility, | 430 |
| Cyanosis, | 54 | Malformation, | 24 |

By this deduction, we reduce the annual city mortality to 10,177, thus making the deaths from disease to population as 1 in every 54, instead of 1 in 44½, and as 18·503, in place of 20·606 in every thousand.

Without the deductions we have proposed, our city will compare for health with any other large city in the United States—the death-rate being less, whether among children or adults.

